# DSENet: Directional Signal Extraction Network for Hearing Improvement on Edge Devices

**DOI:** 10.1109/access.2023.3235948

**Published:** 2023-01-11

**Authors:** ANTON KOVALYOV, KASHYAP PATEL, ISSA PANAHI

**Affiliations:** Department of Electrical and Computer Engineering, The University of Texas at Dallas, Richardson, TX 75080, USA

**Keywords:** Real-time, directional signal extraction, signal separation, beamforming, microphone array

## Abstract

In this paper, we propose a directional signal extraction network (DSENet). DSENet is a low-latency, real-time neural network that, given a reverberant mixture of signals captured by a microphone array, aims at extracting the reverberant signal whose source is located within a directional region of interest. If there are multiple sources situated within the directional region of interest, DSENet will aim at extracting a combination of their reverberant signals. As such, the formulation of DSENet circumvents the well-known crosstalk problem in beamforming while providing an alternative and perhaps more practical approach to other spatially constrained signal extraction methods proposed in the literature. DSENet is based on a computationally efficient and low-distortion linear model formulated in the time domain. As a result, an important application of our work is hearing improvement on edge devices. Simulation results show that DSENet outperforms oracle beamformers, as well as state-of-the-art in low-latency causal speech separation, while incurring a system latency of only 4 ms. Additionally, DSENet has been successfully deployed as a real-time application on a smartphone.

## I. INTRODUCTION

In the past few decades, several signal separation methods have been proposed in the literature for tackling the famous *cocktail party problem*. In the cocktail party problem, we wish to separate the overlapping speech signals, captured by an array of one or more microphones, coming from multiple people talking at the same time. Popular signal separation methods include the use of independent component analysis (ICA) [1], [2], independent vector analysis (IVA) [3], [4], and deep neural networks (DNNs) [5], [6], [7]. Signal extraction is a concept closely connected to signal separation. Unlike signal separation, which, given a signal mixture, aims at extracting all signal sources, signal extraction only extracts an individual target signal. As such, signal extraction is more suitable for hearing improvement applications, where, for efficiency, a single target signal should be extracted and presented in real time. In this work, we are specifically interested in low-latency signal extraction for hearing improvement on edge devices such as smartphones, smart glasses and hearing aids.

Methodology on signal extraction is in general similar to that of signal separation. In signal extraction, however, some type of cue about the source of interest or prior assumption about the mixture are necessary to isolate the target signal from other signals present in the mixture. For instance, Even et al. [8] proposed a method which assumes a dominant target source mixed with diffuse noise created by other less dominant sources. Similarly, Koldovsky et al. [9] assumed a non-Gaussian target source mixed with a Gaussian background. Weng et al. [10] developed a DNN which assumes a mixture of two overlapping speeches and extracts the target based on energy and/or pitch features. Wang et al. [11] proposed a DNN which can extract either female-only or male-only speech from different gender mixtures. Delcroix et al. [12] proposed a DNN capable of tracking an individual speech source in a multi-talker mixture using a set of known voice utterances of the target speaker as a cue. In addition to voice utterances of the target speaker, Xiao et al. [13] further proposed exploiting voice utterances of the competing speakers to improve the DNN’s extraction performance. In a rather different approach, Ephrat et al. [14] proposed a DNN which in addition to a single-channel audio stream takes as input cropped video segments of a localized speaker’s face, allowing to both isolate the speaker of interest and improve extraction performance. Finally, in perhaps the most popular and practical approach, spatial cues are utilized by the various multi-channel signal extraction methods in [15], [16], [17], [18], and [19]. Spatial cues here refer to both knowledge of microphone array geometry and either complete or partial knowledge of relative source locations.

In mulitpath or reverberant environments, source signals are time delayed and convolved. Hence, in the time-domain, the mixing process is modeled as a convolutive mixture. The majority of either signal separation or extraction methods in the literature, including those mentioned above, simplify the mixing model by tackling the problem in the frequency domain using the short-time Fourier Transform (STFT). Using STFT, assuming the window length is sufficiently longer than the mixing filter, convolution in the time-domain is approximately converted to multiplication in the frequency domain. One drawback, however, is that a rather large window length is needed. In fact, a window size of 32 ms is commonly used in literature, resulting in somewhat excessive latency for hearing improvement applications.

In recent years, the work of Luo et al. [20], [21], [22], [23], [24], [25] on DNN-based speech separation in the time-domain has gained a lot of interest in the literature. Time-domain speech separation methods, such as the real-time formulations of the Time-domain Audio Separation Network (TasNet) [20], the fully-convolutional TasNet (Conv-TasNet) [21], and the Filter-and-Sum Network (FaSNet) [25], have shown that time-domain DNNs can achieve high separation performance comparable to frequency-domain approaches, while attaining considerably lower latency. In fact, signal extraction variants of the aforementioned time-domain networks have already been proposed in the literature. For instance, Xu et al. [26] offered the Time-domain speaker extraction Network (TseNet), a DNN conditioned on known voice utterances of a target speaker as cue for extracting a speech signal of interest. Additionally, Gu and Zou [27] proposed the Temporal-Spatial Neural Filter, a multi-channel variant of Conv-TasNet for signal extraction based on spatial cues.

Most edge devices nowadays come equipped with an array of two or more microphones. Microphone arrays are useful in determining the space-time structure of an acoustic field. Thus, as shown in [15], [16], [17], [18], [19], and [27], assuming no spatial ambiguities, the use of a microphone array coupled with spatial cues can often prove sufficient in identifying and extracting a source of interest without the need of either further assumptions about the type of mixture, as in [8], [9], [10], and [11], or direct cues about the target source, as in [12], [13], [14], and [26]. A practical limitation of the spatially constrained signal extraction methods in [15], [16], [17], [18], [19], and [27], however, is the need of precise estimates of source locations, which, with audio-based measurements alone, are especially hard to obtain in multi-talker scenarios unless visual cues are also available. Moreover, it is unclear what happens when the location estimates are not precise and the sources are near each other.

In this work, we propose Directional Signal Extraction Network (DSENet), a real-time, multi-channel signal extraction DNN specifically designed for hearing improvement on edge devices. Given a reverberant mixture, DSENet aims at extracting the reverberant signal, as captured by the reference microphone, whose source is located within a predefined directional region with respect to the microphone array. If multiple sources are located within the directional region of interest, DSENet aims at extracting a linear combination of their reverberant signals. Consequently, when compared to conventional spatially constrained signal extraction approaches, the formulation of DSENet does not require precise estimates of source locations while, at the same time, provides a practical and clearly defined approach for handling spatial ambiguity cases.

Many smartphones nowadays offer a feature known as *audio zoom* [28], [29]. Audio zoom uses spatial filtering, also known as beamforming, to combine the signals captured by the microphone array of the device in such a way to produce a spatial pattern that maximizes the response towards a direction of interest while attenuating the interfering signals located at directions of no interest. In reverberant environments, however, the interfering signals may reach the microphone array from many directions, including the direction of interest, resulting in a problem known as *crosstalk*.^1^ Unlike beamforming, the formulation of DSENet, in principle, circumvents crosstalk, thus providing an alternative approach to audio zoom. Apart from smartphones, DSENet can also be similarly used in wearable devices featuring a microphone array, such as hearing aids or smart glasses, to allow focusing sound capture towards the line of sight of the user.

The proposed DSENet introduces the following five contributions. (1) *Practical signal formulation*: precise estimates of source locations are not required; spatial ambiguity cases are handled in a clearly defined manner; no crosstalk in target signal definition. (2) *Low latency*: extraction is performed directly in the time-domain; a latency of only 4 ms is attained. (3) *Low distortion*: as in FaSNet, a linear signal model based on the conventional beamforming technique of filter-and-sum (FaS) is applied. Additionally, a linear interpolation technique is proposed for smoothing out possible distortions due to time-varying filtering. (4) *Limited computational and memory complexities*: a small and relatively simple network, which can be feasibly deployed on an edge device, is proposed. In fact, DSENet has been successfully implemented on a smartphone. (5) *High performance*: DSENet is shown to significantly outperform both time and frequency domain formulations of oracle^2^ MVDR beamformers in all test metrics. For matching target signal cases, DSENet is also shown to outperform state-of-the-art (SOTA) in low-latency causal speech separation Conv-TasNet and FaSNet models.

The reminder of this paper is structured as follows. The proposed DSENet is introduced in Section II. Experiment configurations are described in Section III. Results are reported in Section IV. Finally, in Section V we conclude the paper and discuss future research.

By convention, vectors in this paper are column vectors. Bold lower case letters denote vectors and bold upper case letters represent matrices. **x**[*i*] is the *i*-th element of **x**. **x**[*i* : *j*] is a subvector formed by the *i*-th through the *j*-th elements. **x**^*T*^ is the transpose of **x**. ‖**x**‖ is the Euclidean norm of **x**. E[⋅] denotes expectation. 𝒰(*a*, *b*) denotes uniform distribution between *a* and *b*. $\left(\hat{\cdot }\right)$ denotes an unknown estimate that needs to be found. FC denotes a fully connected layer. GRU denotes a gated recurrent unit layer. LN denotes layer normalization [32]. PReLU denotes a parametric rectified linear unit activation function [33].

## II. DIRECTIONAL SIGNAL EXTRACTION NETWORK (DSENet)

### A. TASK DEFINITION

**FIGURE 1.**
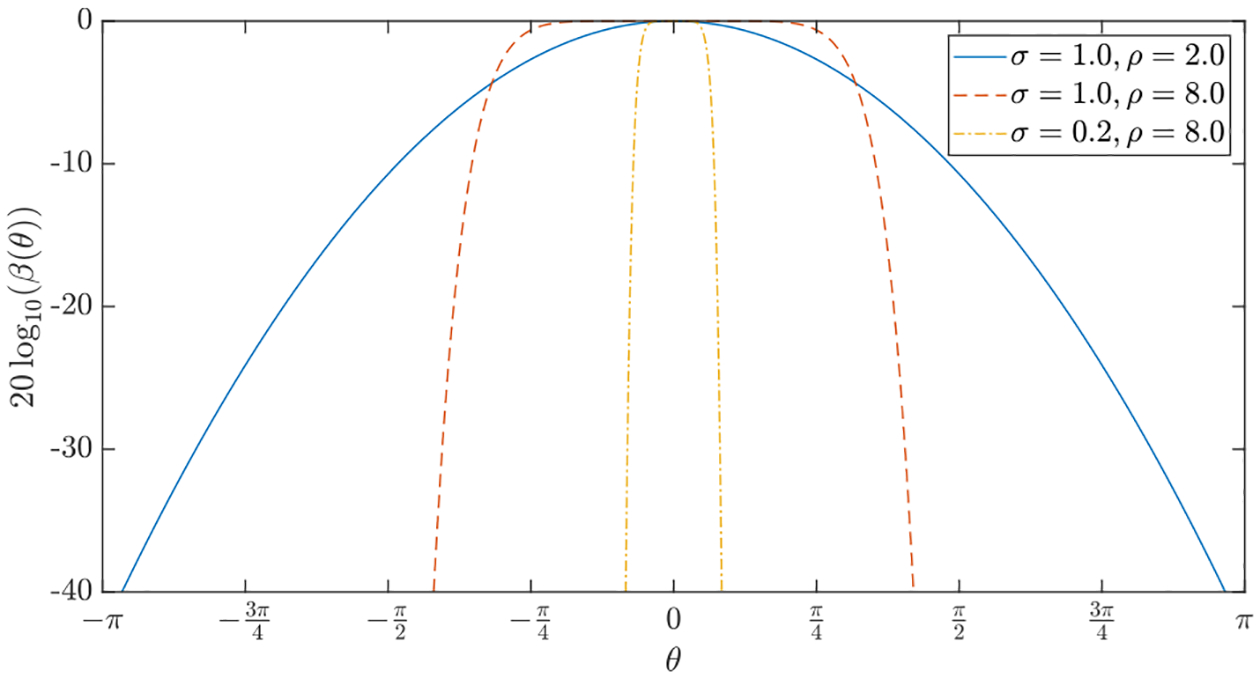
Beampatterns for different combinations of *σ* and *ρ*.

Let us consider a microphone array of *M* elements and arbitrary geometry in a reverberant environment with *N* sources. The time-domain signal captured by the *m*-th microphone is modeled by

(1) $$y_{m}=\overset{N}{\underset{i=1}{\sum }}g_{m,i}*s_{i}\;=\overset{N}{\underset{i=1}{\sum }}x_{m,i},\;m=1,2,\ldots ,M$$

where **g**_*m*,*i*_ is the impulse response of the *i*-th source, **s**_*i*_, with reference to the *m*-th microphone, and **x**_*m*,*i*_ is the resulting reverberant signal. For simplicity, background and internal microphone noises are neglected. Each source is assumed to be at far field from the microphone array. The goal is to extract a linear combination of the reverberant signals, as captured by a reference microphone, whose sources are placed sufficiently near a direction of interest with respect to the local coordinate system (LCS) of the microphone array. Direction is here parametrized by the azimuthal angle *θ*. Let the first microphone be the reference. Consequently, the target signal is defined as

(2) $$z=\overset{N}{\underset{i=1}{\sum }}\beta \left(\theta_{i}\right)x_{1,i},$$

where *θ*_*i*_ is the azimuthal angle of the *i*-th source with respect to the LCS of the microphone array and *β*(*θ*) is a scalar gain given as a function of *θ*. It follows that *β*(*θ*) should be 1 when *θ* is near the direction of interest and 0 otherwise. Since the LCS can be defined in an arbitrary manner, without loss of generality, we can select any direction as the direction of interest. For simplicity, let *θ* = 0 denote the direction of interest. For stable performance, *β*(*θ*) should preferably be a continuous function. Assuming a non-linear microphone array, i.e., no front-back ambiguity [34], *β*(*θ*) is here given by the following beam-like function

(3) $$\beta (\theta )=e^{-\frac{1}{2}{\left(\frac{\theta }{\sigma }\right)}^{\rho }},$$

where *σ* and *ρ* are parameters defining the desired beampattern. As shown in Fig. 1, *σ* controls the beam width, while *ρ* controls the beam sharpness.

### B. LINEAR SIGNAL MODEL

Since nonlinear signal models can create unpleasant distortions to the target signal that are challenging to predict or comprehend, they do not seem to be particularly suitable for hearing improvement applications. Therefore, as in FaSNet, a linear signal model based on the conventional beamforming technique of FaS is preferred instead.

#### 1) FILTER AND SUM (FaS)

Signal extraction is performed by applying a time-varying linear filter to each microphone signal and summing the results as given by

(4) $$\hat{z}[n]=\overset{M}{\underset{m=1}{\sum }}h_{m,n}^{T}y_{m}\left[n-L_{p}:n+L_{f}\right],$$

where **h**_*m*,*n*_ is a non-causal linear filter applied at the *m*-th microphone signal and *n*-th time index, and *ẑ*[*n*] is the corresponding sample of the estimated target signal. It follows that the length of **h**_*m*,*n*_ is 1 + *L*_*p*_ + *L*_*f*_, where *L*_*p*_ and *L*_*f*_ are the respective parameters defining the number of past and future samples of the multi-channel input signal used to estimate the target signal.

#### 2) FILTER INTERPOLATION

Estimation of new filters for every output sample is highly impractical in terms of computational efficiency. Instead, assuming the filters do not vary significantly from one time index to the next, we propose estimating the filters every *L* samples followed by applying linear interpolation to smooth out possible distortions due to sudden filter transitions. Let

(5) $${\hat{z}}_{k}=\hat{z}[(k-1)L+1:kL]$$

denote the *k*-th frame of length *L* of the estimated target signal **ẑ**. Similarly, let

(6) $$y_{m,k}=y_{m}\left[(k-1)L+1-L_{p}:kL+L_{f}\right]$$

denote the *k*-th frame of length *L* + *L*_*p*_ + *L*_*f*_ of the input signal **y**_*m*_. The FaS operation in (4) is now reparametrized as follows

(7) $${\hat{z}}_{k}[i]=\overset{M}{\underset{m=1}{\sum }}h_{m,k,i}^{T}y_{m,k}\left[i:i+L_{p}+L_{f}\right],\;i=1,2,\ldots ,L,$$

where **h**_*m*,*k*,*i*_ denotes the filter applied at the *i*-th sample of the input frame **y**_*m*,*k*_. Let **h**_*m*,*k*_ be the filter estimated at the *k*-th frame. **h**_*m*,*k*,*i*_ is given by applying linear interpolation between **h**_*m*,*k*−1_ and **h**_*m*,*k*_ as follows

(8) $$h_{m,k,i}=h_{m,k-1}+\frac{h_{m,k}-h_{m,k-1}}{L}i.$$

At a given frame index *k*, the input to DSENet is thus **y**_*m*,*k*_, for *m* = 1, 2, …, *M*, and the output is **ẑ**_*k*_. Experimental results have shown that excellent trade-off between computational complexity, latency, and extraction performance can be achieved for *L* = *L*_*p*_ = *L*_*f*_.

With the proposed filter interpolation technique, we found that the additional, commonly applied, smoothing step of overlap-add is not necessary. The overlap-add technique is used in FaSNet. With this step, inference is performed every *L*/2 samples followed by overlap adding adjacent outputs to form the extracted frame **ẑ**_*k*_. The fact that this step is omitted here, not only implies a computational speedup by a factor of two, but also lower system latency by *L*/2 samples.

### C. NETWORK DESIGN

**FIGURE 2.**
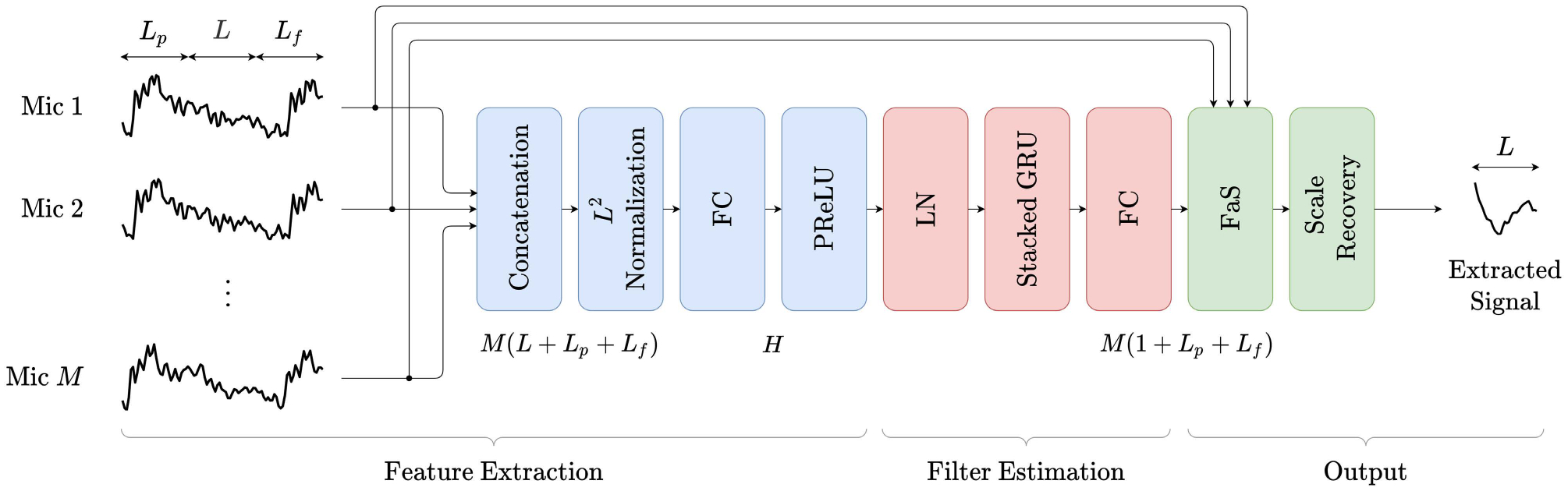
System flowchart of the proposed DSENet model.

As shown in Fig. 2, the architecture of DSENet consists of three processing stages: feature extraction, filter estimation, and output. In the feature extraction stage, the *M* different channel time-domain input frames **y**_*m*,*k*_ in (6) are concatenated to form a vector of length *M*(*L* + *L*_*p*_ + *L*_*f*_) which is then transformed into a lower-dimensional feature vector of length *H*. This lower-dimensional vector is then used in the filter estimation stage to estimate the *M* filters **h**_*m*,*k*_ in (8). In the output stage, the FaS operation in (7) is performed to extract a scaled version of the target signal frame estimate **ẑ**_*k*_ in (5). The scaling comes from the use of a scale-invariant training objective. Thus, an additional scale recovery step is performed in the output stage. FC layers are used to map a given feature space into another of different dimension. Two stacked GRU layers of *H* units each are used to estimate the filters in an intermediate feature space. *H* is set at a low value in relation to the dimension of the input to provide low computational complexity and good scalability for varying microphone array sizes.

#### 1) FEATURE EXTRACTION

Let

(9) $$p_{k}={\left[\begin{array}{c} y_{1,k}^{T}\;y_{2,k}^{T}\;\ldots \;y_{M,k}^{T} \end{array}\right]}^{T}$$

be a vector grouping all *M* different channel input frames **y**_*m*,*k*_. In the feature extraction stage, the vector **p**_*k*_ of length *M*(*L*_*p*_ + *L* + *L*_*f*_) is mapped into a lower-dimensional feature vector **f**_*k*_ of length *H*. In this mapping, **p**_*k*_ is first normalized to have unit *L*^2^ norm to reduce variability. Then, an FC layer is applied followed by a nonlinear activation function to extract **f**_*k*_. The nonlinear activation function used is PReLU. The complete feature extraction procedure is given by

(10) $$f_{k}=\text{PReLU}\left(W\left(\frac{p_{k}}{\left\|p_{k}\right\|+\epsilon }\right)+b\right),$$

where $W\in {\mathbb{R}}^{H\times M\left(L_{p}+L+L_{f}\right)}$ and $b\in {\mathbb{R}}^{H}$ are the respective weight and bias parameters of the FC layer, and *ϵ* = 1e-8 is a constant for numerical stability.

#### 2) FILTER ESTIMATION

Let

(11) $$h_{k}={\left[\begin{array}{c} h_{1,k}^{T}\;h_{2,k}^{T}\;\ldots \;h_{M,k}^{T} \end{array}\right]}^{T}$$

be a vector grouping all *M* different channel filters **h**_*m,k*_ used in (8). In the filter estimation stage **f**_*k*_ is used to estimate **h**_*k*_. This stage consists in first applying LN to ease the training process, followed by two stacked GRU layers of *H* units each, resulting in a vector of length *H*. This vector is then used as input to an FC layer of *M*(*L*_*p*_ + *L*_*f*_ + 1) units to output **h**_*k*_.

#### 3) OUTPUT

In the output stage, currently estimated filters grouped by **h**_*k*_ in (11) are used in (8) along with previously estimated filters grouped by **h**_*k*−1_ to interpolate **h**_*m*,*k*,*i*_ and perform the FaS operation in (7). Due to the use of a scale-invariant training objective, the output, however, is a scaled estimate of the target signal. Thus, an additional scale recovery step is needed. This step is covered separately in Section II-E.

### D. TRAINING OBJECTIVE

Maximization of scale invariant signal to distortion ratio (SI-SDR) [35] is used as the training objective. SI-SDR is a widely popular evaluation metric in signal separation tasks. Here, SI-SDR is defined by

(12) $$\text{ SI-SDR }=10\;{\text{log}}_{10}\left(\frac{∥\alpha z{∥}^{2}}{∥\alpha z-\hat{z}{∥}^{2}+\epsilon }+\epsilon \right),$$

where

(13) $$\alpha =\frac{z^{T}\hat{z}}{∥z{∥}^{2}}$$

is the scalar projection of the estimated signal **ẑ** onto the target signal **z**.

### E. SCALE RECOVERY

Since maximization of SI-SDR is used as the training objective, the model will incur an arbitrary scale on the estimated signal, which we assume is fixed across all input samples regardless of the signal characteristics over time. Let *z* be an arbitrary sample of the target signal. Let *ẑ* be the scaled estimate of *z*. We wish to find a scalar *η* that, when multiplied with any *ẑ*, gives a good approximation of the corresponding target signal. Minimization of the mean squared error (MSE) is used here to estimate *η*, which conveniently gives us the following closed-form solution

(14) $$\hat{\eta }=\underset{\eta }{\text{arg min }}E\left[{\left(\eta \hat{z}-z\right)}^{2}\right]=\frac{E[\hat{z}z]}{E\left[{\hat{z}}^{2}\right]}.$$

It follows that *η* can be estimated offline using a select set of training utterances. To minimize the effect of noise in the estimation of *η*, we select the training utterances for which SI-SDR is maximized. These utterances consist of a single source positioned exactly at the direction of interest, i.e., *θ* = 0, in a non-reverberant environment. DSENet attains high performance in terms of SI-SDR in this kind of scenario due to the problem’s simplicity.

## III. EXPERIMENT CONFIGURATIONS

The performance of DSENet is evaluated in multi-talker scenarios.

### A. DATASET

A dataset using clean speech utterances from LibriSpeech [36] was generated to simulate two overlapping speech signals being captured by a microphone array in a reverberant room. The dataset generated 32768, 4096, 5120, 4-second-long multi-channel utterances for training, validation, and testing, respectively. The signals were sampled at sampling frequency *F*_*s*_ = 16 kHz. For each utterance, the length and width of the room were each drawn from 𝒰(5 m, 10 m), and its height was drawn from 𝒰(2 m, 4 m). The reverberation time was drawn from 𝒰(0.1 s, 0.5 s). The overlapping speech sources were divided into two categories, target and masker. Their positions were defined in terms of range, azimuth angle, and elevation angle, with respect to the LCS of the sensor array. The azimuth angle of the target was drawn from 𝒰(−10°, 10°). The azimuth angle of the masker was drawn from 𝒰(−180°, 180°). The elevation angle of both sources was fixed at 0° and their ranges were each drawn from 𝒰(0.5 m, 2 m). The signal-to-interference ratio (SIR) was drawn from 𝒰(−5 dB, 5 dB). For practical purposes, the 3-element array of nonlinearly and non-uniformly distributed microphones of an actual edge device, i.e., a Pixel 3 smartphone, was used. The 3-dimensional (3D) microphone positions are given in Table 1. These positions are defined with respect to an LCS chosen in a way such that the angle of interest, i.e., *θ* = 0°, is at the top of the device. The LCS of the microphone array was then brought to the middle of the room and the room impulse responses (RIRs) were generated using the image method [37]. The parameters defining the target signal, that is *σ* and *ρ*, were set to 0.2 and 8, respectively.

### B. HYPERPARAMETERS

The model was trained for 100 epochs with a learning rate of 1e-3 and exponential decay of 0.98 every two epochs. Adam [38] was used as the optimization algorithm. The batch size was set to 8. *L* was set to 32 samples, which was chosen according to the minimum burst size of 2 ms in many devices. To ensure a small model size, the number of filter coefficients for each channel was bounded to 2*L* + 1 and *H* was set to 128. Thus, resulting in a model size of roughly 260K parameters. The scale incurred by the model was estimated using a set of 128 randomly generated training utterances. As per Section II-E, these training utterances consisted of a single source positioned at *θ* = 0° and no reverberation.

### C. PERFORMANCE METRICS

The performance metrics used are: signal-to-noise ratio [35] improvement (SNRi), SI-SDR improvement (SI-SDRi), narrowband Perceptual Evaluation of Speech Quality (PESQ) [39], and Short-time Objective Intelligibility (STOI) [40].

## IV. RESULTS

### A. PERFORMANCE FOR VARYING FILTER CONFIGURATIONS

Different network configurations were trained by varying *L*_*p*_ and *L*_*f*_. As shown in Table 2, the noncausal filter configurations achieve excellent tradeoff between performance and latency. It should also be noted that all configurations achieve high SNRi, a scale variant metric, thus indicating the effectiveness of the scale recovery technique. In the remainder of this paper, we use the best performing configuration as per the results in Table 2 with *L*_*p*_ = *L*_*f*_ = *L*. Excluding processing time, this configuration incurs a system latency of (*L* + *L*_*f*_)/*F*_*s*_ = 4 ms.

### B. BENCHMARKING AGAINST MVDR BEAMFORMERS

For reference, DSENet is benchmarked against the well-known MVDR beamformers. There are multiple MVDR formulations in literature, for a fair comparison with DSENet, we consider only those which do not perform dereverberation. Among these MVDR formulations, both time and frequency domain implementations are examined. The time-domain MVDR (TD-MVDR) is based on the formulation in [31] and the frequency domain MVDR (FD-MVDR) is based on [30]. TD-MVDR is parametrized in the same manner as DSENet, i.e., we let *L*_*p*_ = *L*_*f*_ = 32. FD-MVDR, on the other hand, is known to perform best with a larger frame size. Consequently, we include two FD-MVDR configurations, one with a 4 ms frame size (FD-MVDR-4) and the other with a 32 ms frame size (FD-MVDR-32). Both FD-MVDR configurations use Hann windowing and 50% overlap. The second order statistics of desired and interference signals, required by the MVDR beamformers, are estimated using the entire utterances of the actual desired and interference signals prior mixing. Hence, it should be noted that DSENet is benchmarked against oracle MVDR implementations. Finally, due to DSENet’s rather unusual target signal definition in (2) to further ensure a fair comparison, at least for cases in which there is sufficient angular spacing between target and masker, during evaluation, the target position was fixed at 0° and the performance metrics were computed with respect to MVDR’s target signal, that is, the reverberant target signal as captured by the reference microphone.

**FIGURE 3.**
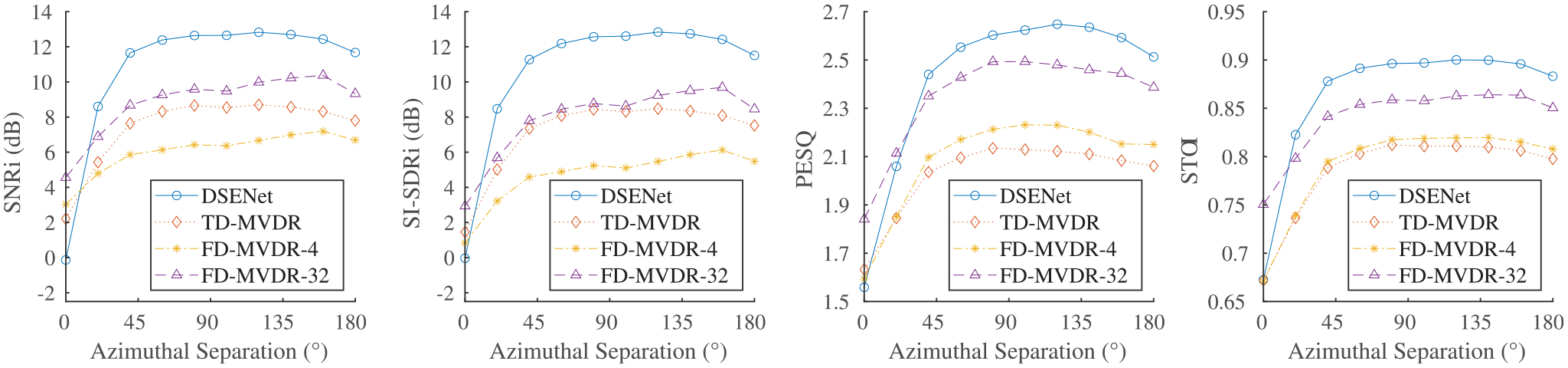
Performance of DSENet versus oracle MVDR beamformers for varying azimuthal separation between target and masker positions.

**FIGURE 4.**
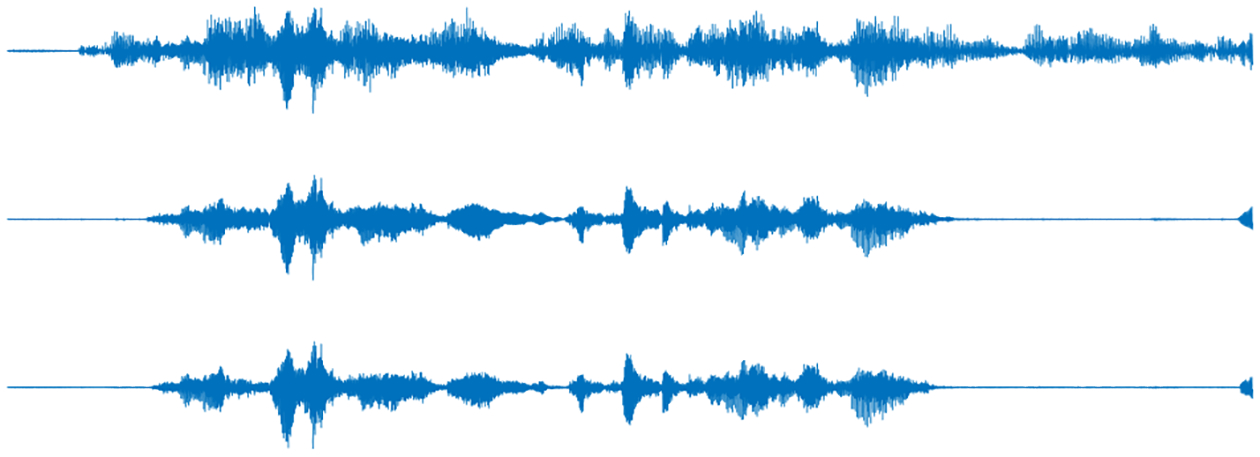
Sample utterance. From top to bottom: mixture, target, and extracted with DSENet waveforms.

Performance results of DSENet with respect to MVDR beamformers for varying angular separation between masker and source are shown in Fig. 3. As per desired behavior, and consistent with the target signal definition in (2), when there is no angular separation between the two speech sources, DSENet does not incur a gain nor loss in either SNR or SI-SDR metrics. This comes from the fact that based on spatial cues alone there is an ambiguity in which signal is the target among the two sources. Hence, the input signal at the reference microphone remains virtually unmodified, resulting in zero gain. In the case of the MVDR beamformers, on the other hand, there is no ambiguity due to oracle knowledge of the different signal statistics. Yet, as expected, performance is still not impressive owing to limited spatial discrimination. However, once there is better spatial discrimination, performance of the different methods improves with DSENet being clearly on top in all metrics. The fact that DSENet outperforms FD-MVDR-32 while attaining much lower system latency is especially remarkable. Using a sample utterance, Fig. 4 further illustrates the extraction capability of DSENet.

### C. COMPARISON WITH SOTA IN CAUSAL SPEECH SEPARATION

When the number and locations of the different sources are available, signal separation methods could in principle tackle the problem in this work in a more general, although less efficient, manner. Hence, it is of interest to verify how DSENet fares in terms of both performance and efficiency with respect to these methods. For this purpose, we compare the signal extraction performance and computational and memory complexities of DSENet with that of SOTA in causal speech separation (CSS). Since the target application is hearing improvement, only low-latency CSS methods are considered, which, as DSENet, have a 2 ms frame size. These include the single-channel Conv-TasNet [21] and the multi-channel FaSNet [25]. Both CSS models were trained on the two-speaker speech separation task without dereveberation. Similar training and validation datasets to those described in Section III-A were generated with the only difference that the azimuth angles of both sources were independently drawn from 𝒰(−180°, 180°) to avoid introducing spatial bias. Conv-TasNet was implemented according to the high-performing causal configuration in [21]. FaSNet, on the other hand, was implemented in the same manner as the causal configuration in [25] with the exception that we increased both the number of input channels in each convolutional block and the embedding dimension from 64 to 80. This was done to compensate for the use of greater sampling rate. Both CSS methods were trained under the same conditions as DSENet described in Section III-B. For a fair comparison, only utterances for which the target signal definition of DSENet overlaps with that of one of the target signals of CSS methods are considered. For this purpose, we employ the same target signal definition and evaluation dataset described in Section IV-B with the exception that utterances for which the azimuthal separation between the sources is below 20° are neglected. The performance metrics of the CSS methods are then computed with reference to the separated reverberant speech signal attaining highest SI-SDR with respect to the reverberant target signal.

Table 3 shows the signal extraction performance and computational and memory complexities of DSENet with respect to the two SOTA models in low-latency CSS. The field MAC/s stands for the number of multiply-accumulate operations per second of a given model when performing inference. SNRi is ignored due to the use of scale invariant task definition in CSS methods. Results show that, at least for matching target signal scenarios, DSENet outperforms SOTA in low-latency CSS in all test metrics while incurring only a small fraction of computational and memory complexities. Despite its massive size, Conv-TasNet attains the worst performance among the three methods, which is not surprising since it is the only single-channel method. The significant performance gain of DSENet over FaSNet, however, was not entirely expected since both methods are multi-channel and follow a FaS approach. This gain can be attributed to the following two factors. (1) DSENet does not generalize as much as FaSNet since it is trained on a more constrained problem, which makes its learning process simpler. (2) DSENet uses a unified approach to estimate the individual-channel filters for the FaS operation, whereas in FaSNet, individual-channel filters are estimated in a partially independent manner to provide invariance to different numbers and locations of microphones, which although of certain practical importance, may weaken the separation performance of the model.

### D. IMPLEMENTATION ON SMARTPHONE

**FIGURE 5.**
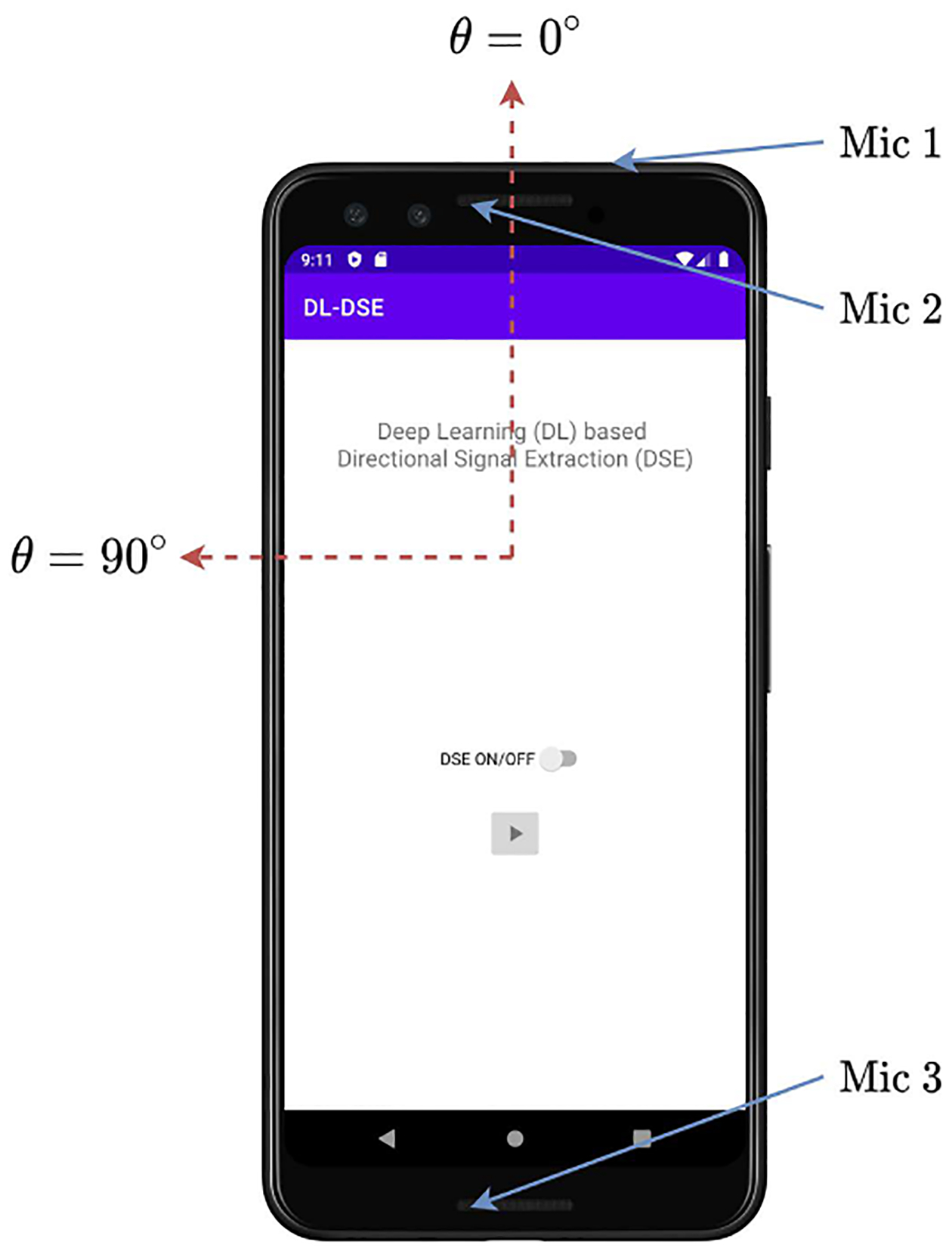
DSENet implemented as a mobile application on a Pixel 3 smartphone. With this application, hearing can be improved, with nearly negligible latency, towards signals originating at a direction of interest by simply pointing the top of the device in that direction.

With the aid of TensorFlow Lite and the Android Native Development Kit (NDK), DSENet was successfully deployed on a Pixel 3 smartphone in the form of a mobile application, i.e., an app. This mobile application is demonstrated in Fig. 5. The per frame processing time was consistently below the 2 ms burst size of the device without the need of post-training quantization or any other computational complexity reduction schemes besides those previously discussed. When tested in the field, we noticed that the implemented model not only generalized well in terms of signal extraction in multi-talker scenarios but, as a positive side effect, also attained noticeable background noise suppression, despite being trained using exclusively speech signals.

## V. CONCLUSION AND FUTURE WORK

This paper proposed DSENet, a network for directional signal extraction using a microphone array. The target signal of DSENet is defined as the linear combination of the reverberant signals, as captured by a reference microphone, whose sources are placed within a directional region of interest with respect to the LCS of the microphone array. As a result, this formulation circumvents the crosstalk problem in beamforming while providing a different and perhaps more practical approach to conventional spatially constrained signal extraction. The primary application of DSENet is hearing improvement on edge devices. As in TaSNet-like systems, signal extraction is performed directly in the time domain. Consequently, the nearly negligible latency of 4 ms is attained. To avoid strange distortions common to DNNs, a linear signal model based on the conventional beamforming technique of FaS is used. Additionally, filter interpolation is proposed to reduce computational complexity and smooth out filter discontinuities. The network architecture of DSENet is relatively simple and, as such, can be easily deployed on an edge device. In fact, DSENet has been successfully implemented on a smartphone. Moreover, despite its small size, when tested on signal extraction in multi-talker scenarios, the developed model is shown to clearly outperform both oracle MVDR beamformers and SOTA in low-latency CSS.

Further research may explore other efficient alternatives to the architecture of DSENet with the aim of improving directional signal extraction performance without excessive compromise on memory and computational complexities. Introducing some degree of dereverberation to the task definition may also be of interest. The aim would be to evaluate the effect of either partial or complete dereverberation on extraction performance, both in terms of target signal distortion and interference signal rejection.

## Figures and Tables

**TABLE 1.** Microphone array 3D positions (cm).

Axis	Sensor 1	Sensor 2	Sensor 3
x	5.1	4.1	−9.2
y	−1.9	0.9	1.0
z	0.0	0.0	0.0

**TABLE 2.** Performance of DSENet for varying *L*_*p*_ and *L*_*f*_.

*L*_*p*_	*L*_*f*_	SNRi	SI-SDRi	PESQ	STOI
2 *L*	0	9.66	9.63	2.42	0.87
*L*	*L*	**10.46**	**10.31**	**2.60**	**0.88**
0	2 *L*	9.97	10.00	2.49	0.88

**TABLE 3.** Comparison with SOTA in causal time-domain speech separation.

Method	Model size	MAC/s	SI-SDRi (dB)	PESQ	STOI
Conv-TasNet	5.07M	5.56B	5.70	1.80	0.77
FaSNet	1.66M	4.12B	9.37	2.15	0.85
DSENet	0.26M	0.13B	**11.85**	**2.52**	**0.88**

## Footnotes

1 Certain adaptive beamformers avoid crosstalk by assuming knowledge of second order statistics of target and/or interference signals, e.g., the minimum variance distortionless response (MVDR) beamformers in [30] and [31] These statistics, however, can be especially hard to estimate in reverberant multi-talker scenarios.

2 Second order statistics required by MVDR are acquired directly from the individual signals prior mixing.

## References

[1] Hyvärinen A and Oja E, “Independent component analysis: Algorithms and applications,” Neural Netw, vol. 13, nos. 4–5, pp. 411–430, Jun. 2000. 10946390 10.1016/s0893-6080(00)00026-5

[2] Zhang K, Wei Y, Wu D, and Wang Y, “Adaptive speech separation based on beamforming and frequency domain-independent component analysis,” Appl. Sci, vol. 10, no. 7, p. 2593, Apr. 2020.

[3] Kim T, Attias HT, Lee S-Y, and Lee T-W, “Blind source separation exploiting higher-order frequency dependencies,” IEEE Trans. Audio, Speech Language Process, vol. 15, no. 1, pp. 70–79, Jan. 2007.

[4] Brendel A and Kellermann W, “Accelerating auxiliary function-based independent vector analysis,” in Proc. IEEE Int. Conf. Acoust., Speech Signal Process. (ICASSP), Jun. 2021, pp. 496–500.

[5] Hershey JR, Chen Z, Le Roux J, and Watanabe S, “Deep clustering: Discriminative embeddings for segmentation and separation,” in Proc. IEEE Int. Conf. Acoust., Speech Signal Process. (ICASSP), Mar. 2016, pp. 31–35.

[6] Liu Y and Wang D, “Causal deep CASA for monaural talker-independent speaker separation,” IEEE/ACM Trans. Audio, Speech, Language Process, vol. 28, pp. 2109–2118, 2020. 10.1109/taslp.2020.3007779 PMC7654633 33178880

[7] Yoshioka T, Wang X, Wang D, Tang M, Zhu Z, Chen Z, and Kanda N, “VarArray: Array-geometry-agnostic continuous speech separation,” in Proc. IEEE Int. Conf. Acoust., Speech Signal Process. (ICASSP), May 2022, pp. 6027–6031.

[8] Even J, Saruwatari H, and Shikano K, “Blind signal extraction based speech enhancement in presence of diffuse background noise,” in Proc. IEEE/SP 15th Workshop Stat. Signal Process, Aug. 2009, pp. 513–516.

[9] Koldovský Z and Tichavský P, “Gradient algorithms for complex non-Gaussian independent component/vector extraction, question of convergence,” IEEE Trans. Signal Process, vol. 67, no. 4, pp. 1050–1064, Feb. 2019.

[10] Weng C, Yu D, Seltzer ML, and Droppo J, “Deep neural networks for single-channel multi-talker speech recognition,” IEEE/ACM Trans. Audio, Speech, Language Process, vol. 23, no. 10, pp. 1670–1679, Oct. 2015.

[11] Wang Y, Du J, Dai L-R, and Lee C-H, “Unsupervised single-channel speech separation via deep neural network for different gender mixtures,” in Proc. Asia–Pacific Signal Inf. Process. Assoc. Annu. Summit Conf. (APSIPA), Dec. 2016, pp. 1–4.

[12] Delcroix M, Zmolikova K, Kinoshita K, Ogawa A, and Nakatani T, “Single channel target speaker extraction and recognition with speaker beam,” in Proc. IEEE Int. Conf. Acoust., Speech Signal Process. (ICASSP), Apr. 2018, pp. 5554–5558.

[13] Xiao X, Chen Z, Yoshioka T, Erdogan H, Liu C, Dimitriadis D, Droppo J, and Gong Y, “Single-channel speech extraction using speaker inventory and attention network,” in Proc. IEEE Int. Conf. Acoust., Speech Signal Process. (ICASSP), May 2019, pp. 86–90.

[14] Ephrat A, Mosseri I, Lang O, Dekel T, Wilson K, Hassidim A, Freeman WT, and Rubinstein M, “Looking to listen at the cocktail party: A speaker-independent audio-visual model for speech separation,” 2018, arXiv:1804.03619.

[15] Khan AH, Taseska M, and Habets EA, “A geometrically constrained independent vector analysis algorithm for online source extraction,” in Proc. Int. Conf. Latent Variable Anal. Signal Separat Cham, Switzerland: Springer, 2015, pp. 396–403.

[16] Li L and Koishida K, “Geometrically constrained independent vector analysis for directional speech enhancement,” in Proc. IEEE Int. Conf. Acoust., Speech Signal Process. (ICASSP), May 2020, pp. 846–850.

[17] Li L, Koishida K, and Makino S, “Online directional speech enhancement using geometrically constrained independent vector analysis,” in Proc. Interspeech, Oct. 2020, pp. 61–65.

[18] Gu R, Chen L, Zhang S-X, Zheng J, Xu Y, Yu M, Su D, Zou Y, and Yu D, “Neural spatial filter: Target speaker speech separation assisted with directional information,” in Proc. Interspeech, Sep. 2019, pp. 4290–4294.

[19] Zhang Z, Xu Y, Yu M, Zhang S-X, Chen L, and Yu D, “ADL-MVDR: All deep learning MVDR beamformer for target speech separation,” in Proc. IEEE Int. Conf. Acoust., Speech Signal Process. (ICASSP), Jun. 2021, pp. 6089–6093.

[20] Luo Y and Mesgarani N, “TaSNet: Time-domain audio separation network for real-time, single-channel speech separation,” in Proc. IEEE Int. Conf. Acoust., Speech Signal Process. (ICASSP), Apr. 2018, pp. 696–700.

[21] Luo Y and Mesgarani N, “Conv-TasNet: Surpassing ideal time–frequency magnitude masking for speech separation,” IEEE/ACM Trans. Audio, Speech, Language Process, vol. 27, no. 8, pp. 1256–1266, Aug. 2019. 10.1109/TASLP.2019.2915167 PMC6726126 31485462

[22] Luo Y, Chen Z, and Yoshioka T, “Dual-path RNN: Efficient long sequence modeling for time-domain single-channel speech separation,” in Proc. IEEE Int. Conf. Acoust., Speech Signal Process. (ICASSP), May 2020, pp. 46–50.

[23] Luo Y, Han C, and Mesgarani N, “Group communication with context codec for lightweight source separation,” IEEE/ACM Trans. Audio, Speech, Language Process, vol. 29, pp. 1752–1761, 2021.

[24] Luo Y, Han C, and Mesgarani N, “Distortion-controlled training for end-to-end reverberant speech separation with auxiliary autoencoding loss,” in Proc. IEEE Spoken Lang. Technol. Workshop (SLT), Jan. 2021, pp. 825–832.

[25] Luo Y, Han C, Mesgarani N, Ceolini E, and Liu S-C, “FaSNet: Low-latency adaptive beamforming for multi-microphone audio processing,” in Proc. IEEE Autom. Speech Recognit. Understand. Workshop (ASRU), Dec. 2019, pp. 260–267.

[26] Xu C, Rao W, Chng ES, and Li H, “Time-domain speaker extraction network,” in Proc. IEEE Autom. Speech Recognit. Understand. Workshop (ASRU), Dec. 2019, pp. 327–334.

[27] Gu R and Zou Y, “Temporal-spatial neural filter: Direction informed end-to-end multi-channel target speech separation,” 2020, arXiv:2001.00391.

[28] Duong NQ, Berthet P, Zabre S, Kerdranvat M, Ozerov A, and Chevallier L, “Audio zoom for smartphones based on multiple adaptive beamformers,” in Proc. Int. Conf. Latent Variable Anal. Signal Separat Cham, Switzerland: Springer, 2017, pp. 121–130.

[29] Nair AA, Reiter A, Zheng C, and Nayar S, “Audiovisual zooming: What you see is what you hear,” in Proc. 27th ACM Int. Conf. Multimedia, Oct. 2019, pp. 1107–1118.

[30] Habets EA, Benesty J, Gannot S, and Cohen I, “The MVDR beamformer for speech enhancement,” in Speech Processing in Modern Communication. Berlin, Germany: Springer, 2010, pp. 225–254.

[31] Bai MR, Ih J, and Benesty J, “Time-domain MVDR array filter for speech enhancement,” in Acoustic Array Systems: Theory, Implementation, and Application. Hoboken, NJ, USA: Wiley, 2013, ch. 7, pp. 287–313.

[32] Ba JL, Kiros JR, and Hinton GE, “Layer normalization,” 2016, arXiv:1607.06450.

[33] He K, Zhang X, Ren S, and Sun J, “Delving deep into rectifiers: Surpassing human-level performance on ImageNet classification,” in Proc. IEEE Int. Conf. Comput. Vis, Dec. 2015, pp. 1026–1034.

[34] Tokgöz S, Kovalyov A, and Panahi I, “Real-time estimation of direction of arrival of speech source using three microphones,” in Proc. IEEE Workshop Signal Process. Syst. (SiPS), Oct. 2020, pp. 1–5. 10.1109/sips50750.2020.9195217 PMC8136617 34026330

[35] Roux JL, Wisdom S, Erdogan H, and Hershey JR, “SDR—Half-baked or well done?” in Proc. IEEE Int. Conf. Acoust., Speech Signal Process. (ICASSP), May 2019, pp. 626–630.

[36] Panayotov V, Chen G, Povey D, and Khudanpur S, “Librispeech: An ASR corpus based on public domain audio books,” in Proc. IEEE Int. Conf. Acoust., Speech Signal Process. (ICASSP), Apr. 2015, pp. 5206–5210.

[37] Allen JB and Berkley DA, “Image method for efficiently simulating small-room acoustics,” J. Acoust. Soc. Amer, vol. 65, no. 4, pp. 943–950, 1979.

[38] Kingma DP and Ba J, “Adam: A method for stochastic optimization,” 2014, arXiv:1412.6980.

[39] Rix A, Beerends J, Hollier M, and Hekstra A, “Perceptual evaluation of speech quality (PESQ)—A new method for speech quality assessment of telephone networks and codecs,” in Proc. IEEE Int. Conf. Acoust., Speech, Signal Process, vol. 2, May 2001, pp. 749–752.

[40] Taal CH, Hendriks RC, Heusdens R, and Jensen J, “An algorithm for intelligibility prediction of time–frequency weighted noisy speech,” IEEE Trans. Audio. Speech. Language Process, vol. 19, no. 7, pp. 2125–2136, Sep. 2011. 10.1121/1.3641373 22087929

